# Longitudinal Modeling of Cardiometabolic Dysfunction in Patients With Type 2 Diabetes Using Item Response Theory

**DOI:** 10.1002/psp4.70349

**Published:** 2026-10-15

**Authors:** Chengli Yu, Hanna Kunina, Gustaf J. Wellhagen, Maria C. Kjellsson

**Affiliations:** ^1^ Pharmacometrics Research Group, Department of Pharmacy Uppsala University Uppsala Sweden; ^2^ Université Paris Cité and Université Sorbonne Paris Nord, Inserm, IAME Paris France

**Keywords:** cardiometabolic dysfunction, item response theory, NONMEM, registry data, type 2 diabetes

## Abstract

Cardiometabolic dysfunction in patients with type 2 diabetes (T2D) is reflected by multiple risk factors and cannot be fully characterized by any single biomarker. Item response theory (IRT) provides a framework for integrating observed risk factors into latent measures of underlying cardiometabolic dysfunction. Routinely collected measurements of systolic and diastolic blood pressure (sBP and dBP), high‐ and low‐density lipoproteins (HDL and LDL), triglycerides (TG), HbA1c, and BMI from 33,396 adults newly diagnosed with T2D were analyzed, with biomarkers categorized into four ordered levels. A two‐dimensional model described the data better than a unidimensional, identifying a blood pressure‐related latent variable (ψ_1_) primarily informed by sBP and dBP, and a metabolic latent variable (ψ_2_) primarily informed by HDL, HbA1c, TG, and BMI, while LDL showed low discrimination on both dimensions. At baseline, age at T2D diagnosis was associated with both latent variables, with opposing effects of 0.02 for ψ_1_ and −0.07 for ψ_2_. Sex was associated with ψ_2_, with women scoring 0.43 units lower than men on the latent variable scale. Longitudinally, ψ_1_ and ψ_*2*_ showed post‐baseline offsets of −0.10 and −0.22, respectively, followed by separately estimated annual slopes of approximately −0.03 year^−1^. HbA1c exhibited a distinct trajectory with an initial post‐baseline improvement followed by deterioration, requiring an item‐specific slope of 0.45 year^−1^. The ψ_2_ contributed modest additional information for all‐cause mortality beyond age and sex. This framework integrated routinely collected biomarkers into continuous latent measures of multidimensional cardiometabolic dysfunction while accounting for differences in their contributions to the latent dimensions.

## Study Highlights

- What is the current knowledge on the topic?
  ○ Metabolic syndrome (MetS) frequently coexists with type 2 diabetes (T2D) and increases cardiovascular disease risk, yet cardiometabolic risk factors are typically assessed individually, and their integrated longitudinal assessment to capture cardiometabolic dysfunction remains limited.
- What question did this study address?
  ○ Can longitudinal IRT integrate routinely collected biomarkers into an assessment of cardiometabolic dysfunction and its progression in patients with T2D?
- What does this study add to our knowledge?
  ○ A two‐dimensional IRT model with a blood pressure‐related dimension and a metabolic dimension (HDL, HbA1c, BMI, and TG) in patients newly diagnosed with T2D characterized complementary components of cardiometabolic dysfunction. HbA1c showed an initial post‐baseline improvement, followed by progressive deterioration, requiring an item‐specific slope on the metabolic dimension to describe its distinct trajectory compared to other metabolic biomarkers.
- How might this change drug discovery, development, and/or therapeutics?
  ○ The framework provides continuous latent measures of cardiometabolic dysfunction, enabling characterization of cardiometabolic profiles in patients with T2D and potentially supporting the longitudinal assessment of disease progression across multiple domains rather than through individual biomarkers alone.

## 1 Introduction

Metabolic syndrome (MetS) is a cluster of interrelated metabolic disorders characterized by the co‐occurrence of obesity, dyslipidemia, hypertension, and insulin resistance [1]. Type 2 diabetes (T2D), which accounts for approximately 90%–95% of all diabetes cases [2], commonly develops in the context of MetS. While MetS substantially increases the risk of developing T2D, its components remain clinically relevant after diabetes onset, as they continue to contribute to cardiovascular risk, with cardiovascular disease (CVD) being the leading cause of excess morbidity and mortality in patients with T2D [3, 4].

In this context, glycated hemoglobin (HbA1c), which remains the primary measure of glycemic control [5] in patients with T2D, provides an incomplete assessment of disease progression and patients' cardiovascular risk. Consequently, effective control over T2D in the presence of MetS requires a comprehensive approach that aims to regulate multiple coexisting risk factors to slow disease progression. This multifactorial perspective considers body mass index (BMI), blood pressure (BP), and lipid levels, which together provide a more comprehensive assessment of the underlying disease [6, 7]. Since the included biomarkers extended beyond the conventional components of MetS in this study, the evaluated underlying condition is referred to as cardiometabolic dysfunction.

Although risk factors for T2D and its complications are well recognized, modeling their shared longitudinal patterns and their relationship with underlying cardiometabolic dysfunction remains limited. Earlier models for assessing outcomes and risks in T2D, such as the NIDDM model [8] and the original UKPDS Outcomes Model (UKPDS‐OM) [9], primarily relied on baseline or updated mean values of risk factors rather than modeling their longitudinal trajectories. More recent models, including the CORE Diabetes Model [10] and UKPDS‐OM2 [11], have introduced time‐varying risk factors to better reflect changes in metabolic control. However, risk factors are generally treated as independent predictors. Large‐scale studies by Höskuldsdóttir et al. [12] and Rawshani et al. [13] evaluated the longitudinal relationships between clinical risk factors and complications in T2D but did not account for correlations or shared variance among cardiometabolic indicators.

To characterize underlying cardiometabolic burden that is not directly observable, latent variable approaches have been used to integrate multiple risk factors into continuous measures of MetS severity. Factor‐analytic methods, particularly confirmatory factor analysis (CFA), have been used to develop continuous MetS severity scores that outperform binary MetS definitions in cardiometabolic risk stratification and account for differences in contributions of individual risk factors across demographic groups [14]. While such approaches offer a useful summary of metabolic burden, they generally prespecify a single latent MetS dimension and derive fixed scoring equations.

Item Response Theory (IRT) is a statistical modeling framework initially developed in education and psychometrics to quantify unobservable traits based on observed item responses [15]. Compared with conventional CFA‐based approaches, IRT directly models the probability of individual response categories as a function of the underlying latent variable and characterizes the relationships between each item and latent construct [16]. IRT has increasingly been applied in healthcare, particularly for diseases characterized by complex, multifactorial processes. In pharmacometrics, IRT models have been adapted to evaluate treatment effects and disease progression using longitudinal clinical outcomes, enabling multiple clinical indicators (i.e., items) to jointly inform latent variables reflecting disease severity or functional status [17, 18, 19, 20]. In clinical practice, cardiometabolic risk factors are commonly interpreted through established risk categories with defined cut‐offs [21, 22]. IRT approaches, especially graded‐response models, are designed to derive several continuous latent constructs from such ordered‐categorical items. In most applications, a unidimensional IRT model is developed, in which items inform a single latent disease dimension that reflects overall disease severity [23]. However, multidimensional IRT allows the latent structure to be evaluated by determining whether the included items reflect multiple, rather than single, dimensions of the underlying disease.

The aim of this study was to characterize underlying cardiometabolic dysfunction and its progression in patients newly diagnosed with T2D using longitudinal IRT modeling of multiple cardiometabolic risk factors.

The objectives were to:

- Characterize the relationship between individual cardiometabolic risk factors and the underlying latent structure in adult patients newly diagnosed with T2D.
- Quantify the longitudinal progression of the latent variables.
- Evaluate the predictive value of the latent variables for all‐cause mortality

## 2 Methods

### 2.1 Data

The data for this study originated from the Swedish National Diabetes Register (NDR) [24] and included retrospective, observational information on adults with T2D who had at least one entry in NDR between 2005 and 2012. This study was approved by the Regional Ethical Review Board, Uppsala, Sweden (No. 2013/399).

Patients diagnosed with T2D within one year of their first entry into NDR were selected for the analysis. Data included the time‐invariant variables of sex and age at diagnosis, and longitudinal measurements of BP (systolic and diastolic; sBP and dBP), low‐ and high‐density lipoproteins (LDL and HDL), triglycerides (TG), HbA1c, and BMI. Only individuals with at least one measurement of these variables were included in the analysis. For the IRT analysis, continuous biomarkers were categorized into four ordered categories (i.e., scores), ranging from 0 to 3, with higher scores representing a greater burden associated with the corresponding biomarker. Categorization was used to provide a common ordinal scale across biomarkers that differed in units, ranges, distributions, and clinical interpretations, enabling their integration within a common IRT framework. The category boundaries were informed by clinical guideline thresholds, treatment targets, and established associations with CVD [25, 26, 27, 28]. Where the available guidance did not provide four categories, intermediate boundaries were introduced to achieve a consistent four‐level structure while preserving ordinality. Table 1 summarizes the applied cut‐offs across the seven items.

**TABLE 1:** Initial categorization of risk factors to serve as items in the item response theory model for T2D.

Item—Risk factor	Category			
	0	1	2	3
Systolic Blood Pressure (sBP, mmHg)^a^	< 120	120–< 130	130–< 140	≥ 140
Diastolic Blood Pressure (dBP, mmHg)^a^	< 70	70–< 80	80–< 90	≥ 90
Low‐density Lipoprotein (LDL, mmol/L)^b^	< 1.8	1.8–< 2.6	2.6–< 4.9	≥ 4.9
High‐density Lipoprotein (HDL, mmol/L)^b^	> 1.6	> 1.3–≤ 1.6	> 1.0–≤ 1.3	≤ 1.0
Triglyceride (TG, mmol/L)^b^	< 1.7	1.7–< 5.6	5.6–< 11.3	≥ 11.3
Glycated Hemoglobin (HbA1c, mmol/mol)^c^	< 42	42–< 48	48–< 58	≥ 58
Body Mass Index (BMI, kg/m^2^)^d^	< 25	25–< 30	30–< 35	≥ 35

^a^ Blood pressure categories informed by American Heart Association definitions [26].

^b^ Lipid categories informed by the 2019 ESC/EAS Guidelines for the management of dyslipidemias [27].

^c^ HbA1c categories informed by 2023 ESC Guidelines for the management of cardiovascular disease in patients with diabetes [25].

^d^ BMI categories based on WHO guidelines [28].

### 2.2 IRT Modeling

The IRT modeling was divided into four steps: (i) determine the appropriate number of dimensions, (ii) characterize the item characteristic functions (ICFs) for the items informing the latent variables of the baseline data, (iii) characterize the longitudinal change in the latent variables, and (iv) evaluate predictive performance of the latent variables for all‐cause mortality.

#### 2.2.1 Dimensionality Assessment

The number of latent dimensions was assessed in the *mirt* package by fitting baseline IRT models with all items allowed to inform one to seven dimensions. The models were compared with the unidimensional model using the Akaike information criterion (AIC) and Bayesian information criterion (BIC), with the optimal number of dimensions selected based on the reduction in both criteria.

#### 2.2.2 Baseline Model

In this work, category‐specific response probabilities were determined for subject *i* and item *j*, and the probability (*P*) of observing a score of at least *s* (from 0 to 3) was modeled within a graded‐response model (GRM) framework [15] (Equation 1):

(1) $$P\left(y_{\mathrm{ij}}\geq s\right)=\;\frac{1}{1+e^{\sum_{d=1}^{D}\alpha_{\mathrm{dj}}\cdot \left(b_{j,s}-\psi_{\mathrm{di}}\right)}}$$

where *D* is the number of latent dimensions, $\psi_{i}$ is a normally distributed latent variable (mean = 0 and variance = 1) that represents underlying cardiometabolic dysfunction, with higher values indicating greater dysfunction, and *α*_*dj*_, and *b*_*j,S*_ are the discrimination and difficulty parameters for item *j*, respectively. The discrimination parameter *α*_*dj*_ is specific to dimension *d*, with a higher value indicating a stronger relationship between the item and the dimension. The difficulty parameter *b*_*j,s*_ is specific to score category *s*, with a higher value indicating that a higher latent‐variable value is required to reach the corresponding category score. The probability of each score category was obtained from the cumulative probabilities (Equation 2) [15]:

$$P\left(y_{\mathrm{ij}}\geq 0\right)=1$$

(2) $$P\left(y_{\mathrm{ij}}=s\right)=P\left(y_{\mathrm{ij}}\geq s\right)-P\left(y_{\mathrm{ij}}\geq s+1\right)$$

$$P\left(y_{\mathrm{ij}}\geq S_{j}+1\right)=0$$

A limited covariate analysis was performed. Covariate effects were applied to the latent variables rather than to individual items, so that effects were shared across all items contributing to a given dimension. For the continuous covariate age at T2D diagnosis, a linear additive relationship was used, while for the binary covariate sex, it was additive [29].

#### 2.2.3 Longitudinal Model

The longitudinal model was developed by fixing the baseline item parameters at their final estimates and describing changes over time through the latent variables. For each biomarker, separate post‐baseline discrimination and difficulty parameters were evaluated individually and retained when they resulted in a reduction in objective function value (OFV) and an improvement in the corresponding item‐level visual predictive check (VPC). The time‐varying latent variable $\psi \left(t\right)$ for subject *i* was described by a linear model [15, 17] (Equation 3):

(3) $$\psi_{i}\left(t\right)=\psi_{i0}+\text{slope}\cdot t+\delta$$

where $\psi_{i0}$ is the subject‐specific latent variable at baseline (including covariate effects), slope is the annual change in the latent variable, *t* is time in years, and δ is a post‐baseline (*t* > 0) offset parameter, accounting for the step change in the latent variable shortly after diagnosis. The random effects on both latent variables were retained in the longitudinal model, while additional inter‐individual variability (IIV) was investigated on the slope, poste‐baseline offset, and item‐specific parameters.

### 2.3 Survival Analysis

The data included information on follow‐up time, all‐cause mortality, and administrative right‐censoring, enabling a time‐to‐event (TTE) analysis [30]. All‐cause mortality was described using a Gompertz hazard model [31] with sex‐specific scale ($\lambda_{s}$) and shape ($\gamma_{s}$) parameters (Equation 4).

(4) $$h_{i}\left(t\right)=\lambda_{\mathrm{si}}\cdot e^{\gamma_{\mathrm{si}}\cdot \left({\text{BAGE}}_{i}+t\right)+\sum_{d=1}^{D}\beta_{d}\psi_{d,i}\left(t\right)}$$

where ${\text{BAGE}}_{i}$ is age at T2D diagnosis, $\psi_{d,i}\left(t\right)$ is the time‐varying latent variables, and $\beta_{d}$ is an estimated effect on the mortality hazard, *D* is the number of latent dimensions.

The analysis was performed sequentially, with parameters of the final longitudinal IRT model fixed and the time‐varying latent variables evaluated as mortality predictors. Because the latent variables changed during the follow‐up, the cumulative hazard was obtained by integrating $h_{i}\left(t\right)$ over time. The additional predictive value of the latent variables beyond age and sex was assessed by comparing the base survival model with models in which $\psi_{1}$, $\psi_{2}$, or both were included. The predictive performance of the latent variables was evaluated using the concordance index (C‐index) and Brier score [32]. Together, these two complementary metrics capture overall predictive performance: the ability to rank patients by mortality risk (C‐index), with higher values indicating better ability, and the difference between actual and predicted mortality probability (Brier score), with lower values indicating smaller prediction error.

### 2.4 Software

NONMEM version 7.5.1 [33] was used for model development through Pearl‐speaks‐NONMEM (PsN) version 5.3.1 [34]. All data management was performed in R version 4.3.3 [35]. The dimensionality assessment was conducted using the *mirt* version 1.31 [36] and the *piraid* [37] packages.

### 2.5 Model Selection and Diagnostics

For hierarchical or nested models, the likelihood ratio test (LRT) was used, based on the difference in OFV between two models. Statistical significance was assessed at *p* < 0.05, with the required reduction in OFV (ΔOFV) depending on the number of additional estimated parameters (e.g., ΔOFV < −3.84 for 1 df). The same significance criterion was used for covariate testing at baseline. Selection of non‐hierarchical models during model development was based on the AIC, with lower AIC values driving the selection process.

Item‐specific model fit was assessed using Item Characteristic Curves (ICCs) and Item Information Curves (IICs). Additionally, simulation‐based diagnostics were performed as item‐level categorical VPCs, in which the observed proportion in each score category over time was compared with the corresponding 95% confidence interval (CI) from the simulations. The overall final model's performance was evaluated using a Percentage Score VPC of the cardiometabolic dysfunction (Equation 5):

(5) $$\begin{array}{c} \text{Percentage Score}=\left(\frac{\sum_{i=1}^{n}{\text{Score}}_{i}}{n\times m}\right)\times 100 \end{array}$$

where i indicates an individual item, *m* is the maximum item score, and *n* is the total number of items observed at a given visit.

## 3 Results

### 3.1 Data

The final dataset contained 33,396 patients with 915,218 longitudinal measurements across all 7 risk factors, of which 198,532 were collected at baseline. Among all the items, HbA1c had the most comprehensive records, with all patients being observed at least once. On average, patients had 4.9 (range 1–64) longitudinal records over 2.6 (range 0.01–7.95) years. Mean age at T2D diagnosis was 61.3 years (SD 12.1), and 45.3% were women. During follow‐up, 1550 patients died (4.6%).

The distributions of baseline and longitudinal scores for the items are shown in Figure 1A,B, respectively than 25% of individuals fell into the highest score category (score 3) for sBP, HDL, and HbA1c. In contrast, LDL and TG had many individuals in lower‐score categories. Overall, the proportion of individuals in the highest score category (score 3) decreased slightly over time, except for HbA1c, which initially decreased during the first year, followed by an increase in higher scores, indicating worsening glycemic control. Changes in dBP and LDL over time were slow and showed a modest shift from score 2 to score 1, while other items, such as BMI and sBP, showed an initial small decrease and thereafter remained stable over time. Since very few observations were classified as scores 2 or 3 for TG, they were combined into a single extreme category, resulting in a three‐category model.

**FIGURE 1:**
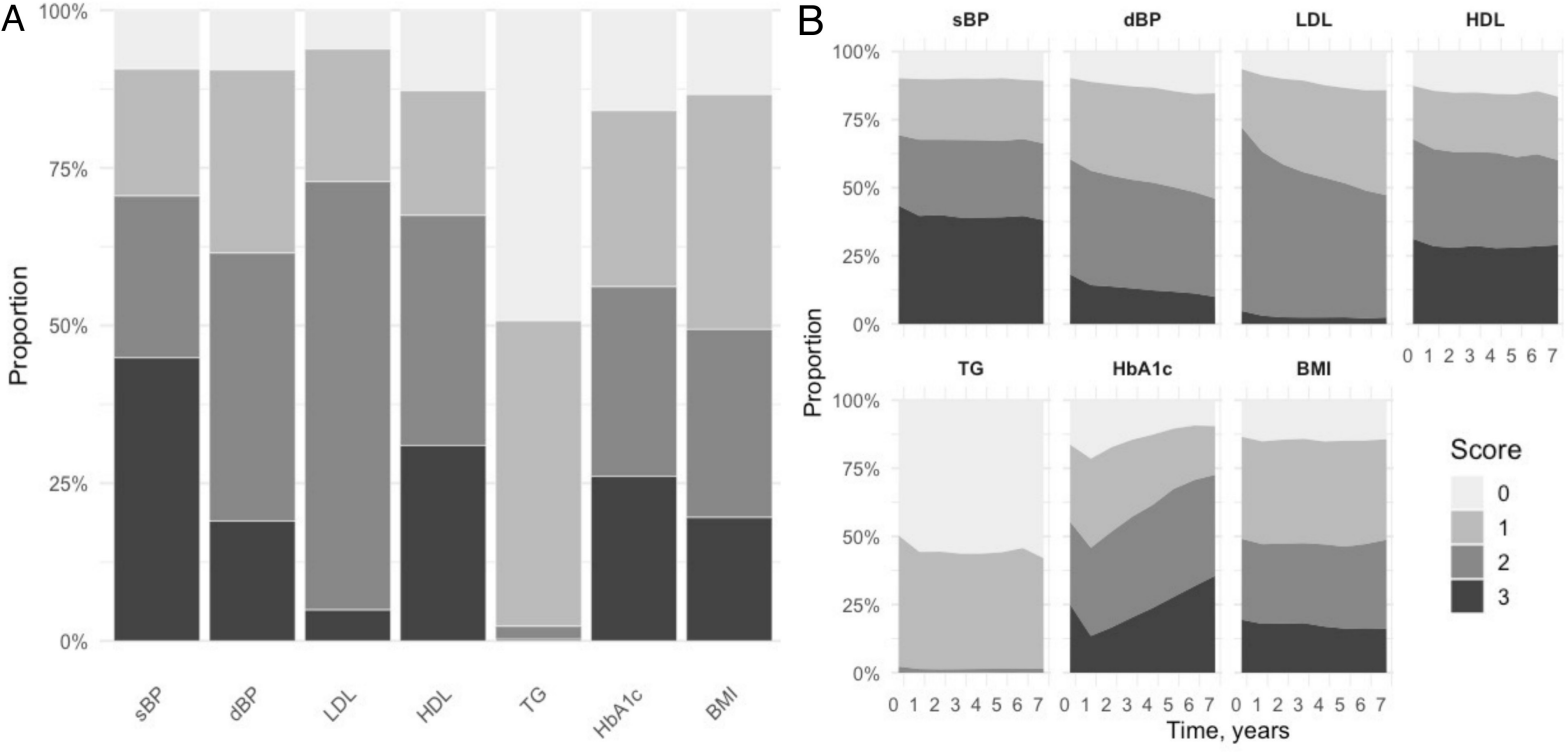
Distribution of scores for all items at baseline (A) and over time (B). Items: sBP—systolic blood pressure, dBP—diastolic blood pressure; LDL—low‐density lipoproteins, HDL—high‐density lipoproteins, TG—triglycerides; HbA1c—glycated hemoglobin; BMI—body‐mass index; ψ_1—_BP‐related latent composite, ψ_2_—metabolic latent composite. Completeness of the items: (A) sBP—96%, dBP—96%, LDL—70%, HDL—73%, TG—73%, HbA1c—100%, BMI—89%; (B) sBP—90%, dBP—90%, LDL—62%, HDL—63%, TG—64%, HbA1c—100%, BMI—86%.

### 3.2 IRT Model

#### 3.2.1 Dimensionality Assessment

The optimal number of latent dimensions was determined by comparing AIC and BIC values across models with one to seven dimensions. Both criteria showed a substantial drop from one to two dimensions (ΔAIC and ΔBIC, both approximately −6000), with minimal further improvement when increasing the number of latent variables beyond two (Figure S1). Consequently, two dimensions were used in the subsequent multidimensional graded‐response IRT (MIRT) model.

#### 3.2.2 Baseline Model

The final baseline MIRT model consisted of seven items, each with two discrimination and three difficulty parameters, except for TG, which had two difficulty parameters, and BMI, which additionally included an item‐specific intercept parameter. The covariates for the baseline model were age at T2D diagnosis on both latent variables (ψ_1_ andψ_2_), and sex on the second latent variable (ψ_2_). The ICCs for the cumulative probabilities of the respective items are shown in Figure 2. Model‐predicted category probabilities showed acceptable agreement with GAM smooths of the observed data for the informative item‐dimension combinations (Figure S3).

**FIGURE 2:**
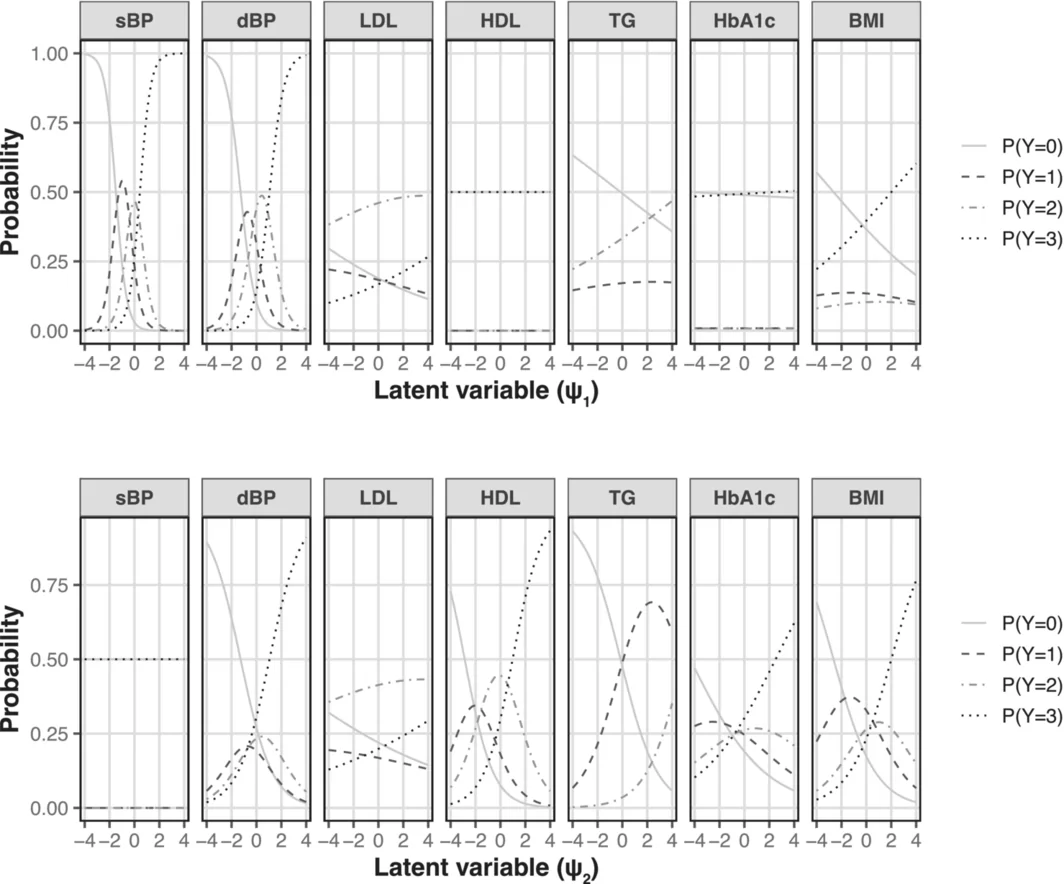
Item Characteristic Curves (ICCs) for all items from the baseline graded‐response MIRT model. Each panel shows the model‐predicted probability of falling into each of the four ordered categories (from 0 to 3) as a function of $\psi_{1}$(blood pressure‐related latent variable; top panel) and $\psi_{2}$(metabolic latent variable; bottom panel). BMI, body‐mass index; dBP, diastolic blood pressure; HbA1c, glycated hemoglobin; HDL, high‐density lipoproteins; LDL, low‐density lipoproteins; sBP, systolic blood pressure; TG, triglycerides.

On ψ_1_, sBP and dBP were the strongest contributing items, with increasing ψ_1_ associated with a higher probability of belonging to higher BP categories. LDL, TG, and BMI showed low discrimination on this dimension, while HDL and HbA1c showed almost none. On ψ_2_, sBP was nearly flat, whereas HDL showed clear changes in category probabilities across the metabolic dimension, indicating its contribution to ψ_2_. In the IICs (Figure 3), sBP and dBP were the most informative items for ψ_1_, while HDL, TG, BMI, HbA1c and dBP were informative for ψ_2_. Baseline model discrimination parameter estimates are presented in Table 2. The difficulty parameters for all items are presented in Table S6.

**FIGURE 3:**
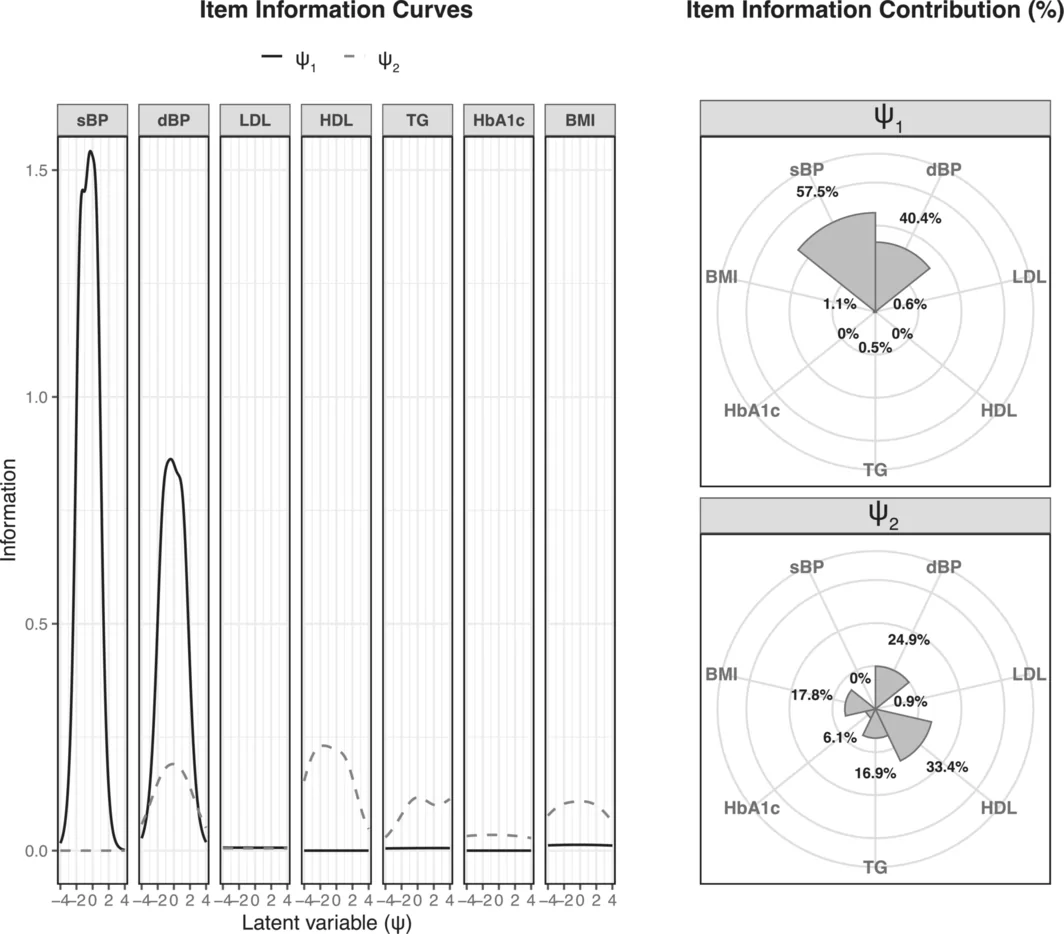
Item information plots for all items for the baseline graded‐response MIRT model. Left panel: Fisher Item Information Curves (IICs) plot, showing how much information each item contributes to $\psi_{1}$ (blood pressure‐related latent variable; solid line) and $\psi_{2}$ (metabolic latent variable; dashed line) on the latent scale. Right panel: item information contribution for $\psi_{1}$ (blood pressure‐related latent variable; top sub‐panel) and $\psi_{2}$ (metabolic latent variable; bottom sub‐panel), expressed as a percentage (%) of the total information per dimension. BMI, body‐mass index; dBP, diastolic blood pressure; HbA1c, glycated hemoglobin; HDL, high‐density lipoproteins; LDL, low‐density lipoproteins; sBP, systolic blood pressure; TG, triglycerides.

**TABLE 2:** Parameter estimates for the baseline and longitudinal IRT model.

Parameter	$\psi_{1}$ Estimate (RSE%)	$\psi_{2}$ Estimate (RSE%)
Baseline IRT model parameters		
Item discrimination parameters		
sBP	2.22 (1.7)	0 (FIX)
dBP	1.84 (2.2)	0.82 (2.8)
LDL	0.18 (12.7)	0.13 (13.8)
HDL	0 (FIX)	0.86 (6.1)
TG	0.12 (20.7)	0.66 (7.9)
HbA1c	0.10 (13.9)	0.33 (6.2)
BMI	0.17 (9.2)	0.59 (3.7)
Covariate effects^a^		
Age at T2D diagnosis, year^−1^	0.02 (4.9)	−0.07 (5.1)
Women versus men	—	−0.43 (6.3)
Longitudinal IRT model parameters		
Item discrimination parameters^b^		
LDL	0.29 (7.7)	0.001 (3.4)
HDL	0 (FIX)	2.53 (3.0)
HbA1c	0.14 (18.9)	0.40 (3.9)
Latent variable parameters		
Slope, year^−1^	−0.028 (9.1)	−0.029 (9.3)
Post‐baseline offset	−0.10 (7.6)	−0.22 (6.2)
Slope_HbA1c_, year^−1^	—	0.45 (4.6)

^a^ Age at T2D diagnosis was centered at mean age at diagnosis (61 years). Men were used as the reference group for the sex covariate.

^b^ Re‐estimated post‐baseline.

sBP and HDL each discriminated primarily on a single dimension (ψ_*1*_ and ψ_*2*_, respectively), while dBP had substantial discrimination on both. LDL had low discrimination on both dimensions. Age at T2D diagnosis was associated with both dimensions, with a positive effect on ψ_*1*_ (0.02 year^−1^), and a negative effect on ψ_*2*_ (−0.07 year^−1^). Inclusion of age at diagnosis improved the model fit (ΔOFV = −4249), while ICCs remained unchanged. Biological sex was associated with ψ_*2*_, with women scoring, on average, 0.43 units lower than men on the latent variable scale. With the random‐effect variance for each latent dimension fixed to 1, the covariate effects can be interpreted relative to one SD of the IIV on the latent variable scale.

#### 3.2.3 Longitudinal IRT Model

To account for longitudinal dynamics, the IRT model was extended by fixing the baseline item‐specific discrimination and difficulty parameters at their final estimates while allowing the latent variables to change over time. The baseline parameters for sBP, dBP, TG, and BMI were retained, whereas for HbA1c, LDL, and HDL, separate post‐baseline discrimination and difficulty parameters were estimated, improving both the model fit and corresponding item‐level VPCs. The longitudinal model parameters with corresponding RSEs (%) are presented in Table 2. Item‐specific re‐estimated difficulty parameters are presented in Table S6.

For HbA1c, this improvement from post‐baseline parameter re‐estimation was insufficient to capture the observed longitudinal pattern. An HbA1c‐specific slope on ψ_2_ (${\text{slope}}_{2,\mathrm{HbA}1c}$ = 0.45 year^−1^) was introduced, replacing the shared slope in Equation (3) for HbA1c observations, further improving the VPC and adequately describing the observed initial improvement followed by subsequent deterioration (Figure 4). The post‐baseline offsets (−0.10 for ψ_1_; −0.22 for ψ_2_) and negative yearly progression rates (−0.03 year^−1^ for both ψ_1_ and ψ_2_) indicated a general decline in latent‐variable scores over time. A stable model could not be identified when including IIV on slopes and post‐baseline offsets, therefore, they were estimated without IIV. Categorical VPCs for each item over time since T2D diagnosis are shown in Figure S2.

**FIGURE 4:**
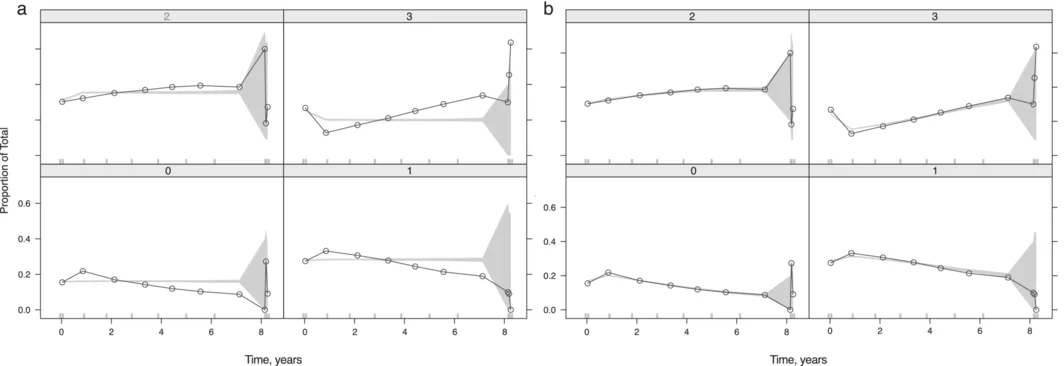
Visual predictive check (VPC) of HbA1c with the common shared ${\text{slope}}_{2}$ (left panel, A) and with the separate ${\text{slope}}_{2,\mathrm{HbA}1c}$ (right panel, B). Each sub‐panel shows the model‐predicted probability of falling into each of the four ordered categories (i.e., 0—bottom left, 1—bottom right, 2—top left, and 3—top right). Blue‐shaded areas represent the 95% CI from model simulations, and the median of the observed data is shown as blue open circles connected by splines.

All items showed an early transition from higher to lower score categories, followed by a slow decline in scores, except for HbA1c, which showed a steep increase in higher score categories over time. Towards the end of the follow‐up period, the sparsity of remaining observations resulted in greater fluctuations in the observed proportions and wider CIs.

Overall final model performance was assessed using the Percentage Score VPC (Figure 5).

**FIGURE 5:**
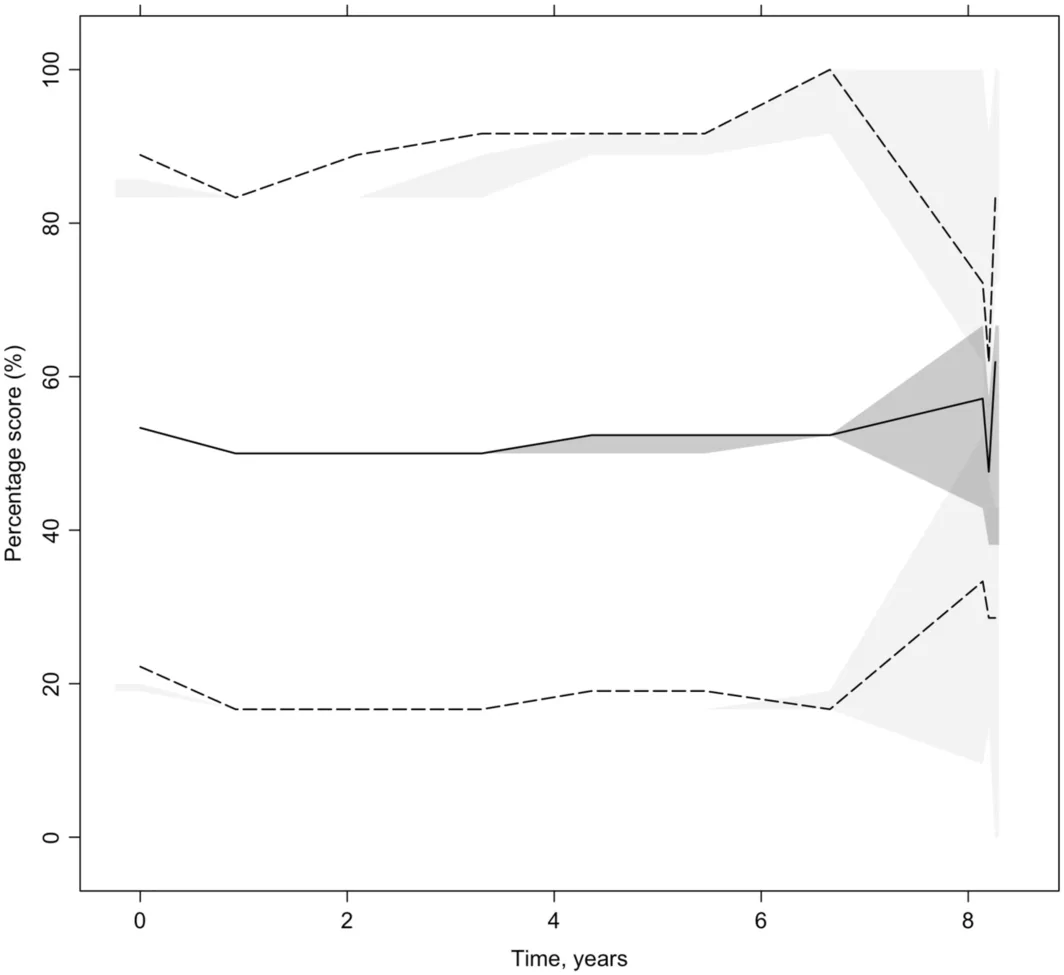
Percentage Score VPC of the final longitudinal MIRT model for the full study population. The lines correspond to the 97.5th (dashed), 50th (solid), and 2.5th (dashed) percentiles of the observed data. The shaded areas correspond to the 95% CIs of the corresponding percentiles of the simulated data.

The Percentage Score VPC evaluated the model's ability to describe overall cardiometabolic burden across all items simultaneously. The median percentage score in this patient population was approximately 50%, with a notable decrease observed in the first year after T2D diagnosis. Observed and simulated medians and 2.5th and 97.5th percentiles overlapped with narrow CIs, indicating that the model adequately described the overall cardiometabolic dysfunction over time. Towards the end of the follow‐up period, as in Figure 4, the wider CIs reflect the sparsity of remaining observations.

### 3.3 Survival Analysis

The performance of the latent variables as mortality predictors was assessed using the LRT, C‐index, and Brier score (Table S4). Both ψ_1_ and ψ_2_ improved the model fit when evaluated individually, with ΔOFV = −2028 and ΔOFV = −1145, respectively (1 df). The reference model without latent variable covariates had a C‐index of 0.672. Adding ψ_1_ alone did not meaningfully improve discrimination (ΔC = 0.001), while the improvement from ψ_2_ was marginally larger (ΔC = 0.015), indicating a modest improvement in ability to correctly rank patients according to their mortality risk. The Brier score showed a very small reduction of 0.0005 attributed to the ψ_2_ inclusion. Including both latent variables simultaneously did not improve predictive performance beyond ψ_2_ alone, based on the Brier score and C‐index.

## 4 Discussion

In this study, a graded‐response MIRT model was developed to characterize cardiometabolic dysfunction in patients with T2D using data from the Swedish NDR. In the unidimensional baseline model, all cardiometabolic risk factors were assumed to contribute to a single shared latent variable. In this model, blood pressure contributed the greatest amount of item information, whereas the remaining items were substantially less informative (Figure S5). This did not adequately represent the multifactorial nature of cardiometabolic dysfunction, driven by cardiometabolic dysregulation rather than hypertension alone. The two‐latent‐variable MIRT model demonstrated a substantial improvement in describing the data compared to the unidimensional model, allowing the biomarkers to inform one or both dimensions to different extents, better representing the latent structure. Consequently, two latent dimensions identified at baseline were retained in the longitudinal model, each representing complementary components of cardiometabolic dysfunction. Additionally, the relative contribution of biomarkers to the latent dimensions was estimated, providing information on the underlying dimensional structure that is not captured by a more common unweighted MetS component count.

The first dimension (ψ_1_) was primarily influenced by BP measurements, with higher sBP and dBP values corresponding to greater BP‐related burden. The second dimension (ψ_2_) reflected broader metabolic dysfunction, with contributions from HDL, TG, BMI, and HbA1c. LDL showed low discrimination on both dimensions, which may partly result from lipid‐lowering treatment‐to‐target, potentially reflecting limited inter‐individual variability of LDL in the treated population rather than a lack of relevance. Retaining weakly informative items introduces additional parameters that provide only limited improvement to the final model. However, as the aim was to characterize cardiometabolic dysfunction using established routinely collected biomarkers, the relative contribution of each of them was considered an informative finding. In contrast to LDL, dBP contributed to both dimensions, suggesting its relevance to both BP‐related and metabolic‐related dimensions [38].

Age at T2D diagnosis and sex were included as covariates in the baseline model. Sex was associated with ψ_2_, with women, on average, scoring 0.43 units lower than men on the latent variable scale. This may partly reflect the use of sex‐independent thresholds for item categorization. Sex‐specific thresholds, if applied, could account for sex‐dependent factors [39, 40], potentially reducing the estimated sex effect. The effects of age at T2D diagnosis also differed between the latent dimensions. For ψ_1_ a positive association was consistent with a greater BP‐related burden corresponding to higher disease severity in patients diagnosed at an older age. The negative association for ψ_2_ suggested that younger age at T2D diagnosis was associated with higher values of the metabolic latent dimension. This potentially reflects a more adverse metabolic phenotype among patients who develop T2D earlier in life [41], or survivor bias, where older patients may represent a healthier subgroup in the current population.

The longitudinal model indicated that applying a shared slope across all metabolic items resulted in a near‐zero estimate, suggesting minimal change over time in the second latent dimension. However, introducing a separate slope for HbA1c resulted in a more strongly negative shared slope for the remaining items, indicating a modest decrease in ψ_2_ over time. Despite its limited discrimination on ψ_2_ at baseline compared to other metabolic biomarkers, the need to model HbA1c separately underscores its distinct longitudinal trajectory. The initial improvement in HbA1c scores was captured by the negative post‐baseline offset, potentially reflecting antihyperglycemic treatment initiation. However, this was transient and followed by a deterioration, with HbA1c worsening more rapidly than other items, reflected by the positive item‐specific ${\text{slope}}_{\mathrm{HbA}1c}$. The dimensionality assessment was performed on baseline data, so whether a third latent dimension, potentially informed by HbA1c, would be supported was not evaluated. Additionally, the longitudinal IRT model demonstrated a modest early post‐baseline offset in ψ_1_, while ψ_2_ showed a change more than twice as large (−0.10 vs. −0.22), indicating greater decrease in metabolic dysfunction. This greater shift in ψ_2_ may reflect improvements in metabolic biomarkers following diagnosis and initiation of antihyperglycemic treatment.

The survival analysis was performed to link the latent dimensions to a clinical outcome, assessing the prognostic value of the developed approach. ψ_1_ did not show an association with all‐cause mortality, whereas the association of ψ_2_, although statistically significant, was modest, which may partly reflect the low mortality in the study population (4.6%). Since the Gompertz hazard model was parameterized to account for age and sex, the improvement from ψ_2_ indicated that the latent variable captured some additional information beyond standard demographics. The small improvement was shown across both the C‐index and Brier score, supporting this conclusion.

Several limitations of this study should be considered. Although the MIRT model was developed using registry data with a large sample size and broad coverage of clinical biomarkers, reliance on routinely collected observational data introduces challenges with data quality and consistency, while the large sample size may make even minor differences statistically significant, despite limited clinical relevance [42]. Furthermore, patients with more severe disease states may have more frequent observations, leading to an overrepresentation of severe disease states and introduction of informative observation bias [43]. The GRM used in the developed IRT framework models ordered categorical item data, requiring the initially continuous biomarker measurements to be categorized, which resulted in part of the information being discarded. Categorization positioned heterogeneous continuous biomarkers on a common, ordinal scale; however, it reduced sensitivity to longitudinal changes occurring within the defined categories. A continuous‐response IRT approach [44], which could retain continuous biomarker data but would introduce additional modeling assumptions and increase model complexity, was not evaluated in the current study. While the probabilistic framework of the MIRT model described longitudinal progression of cardiometabolic dysfunction by capturing variability across individuals and biomarkers, the latent variables remain abstract constructs. Although their associations with all‐cause mortality were assessed, they were not directly linked to cardiometabolic complications, limiting clinical interpretation [15].

To our knowledge, this study is the first to apply MIRT to routinely collected biomarkers for investigating cardiometabolic dysfunction in patients newly diagnosed with T2D. The findings demonstrate that cardiometabolic dysfunction can be characterized longitudinally using multiple biomarkers. Two complementary latent dimensions were identified, with the relative contribution of each biomarker quantified. Further investigations should focus on the clinical interpretation of each latent dimension through its associations with specific clinical outcomes. In conclusion, these findings support the application of MIRT to capture the longitudinal dynamics of cardiometabolic dysfunction and emphasize the relevance of integrated assessment of cardiometabolic risk in patients newly diagnosed with T2D.

## Author Contributions

C.Y., H.K., G.J.W., and M.C.K. wrote the manuscript. C.Y. and M.C.K. designed the research. C.Y. and H.K. performed the research. C.Y. and H.K. analyzed the data.

## Funding

This work has been supported by financial grants from the Swedish Research Council (Dnr 2018‐03316).

## Conflicts of Interest

The authors declare no conflicts of interest.

## Supporting information


**Figure S1:** Change in Akaike Information Criterion (AIC) and Bayesian Information Criterion (BIC) relative to the unidimensional model for baseline IRT models with one to seven latent dimensions. The dashed line at zero represents the reference unidimensional model.
**Figure S2:** Categorical Visual Prediction Checks (VPCs) for the final longitudinal MIRT model. Each sub‐panel shows the model‐predicted probability of falling into each of the four ordered categories (i.e., 0—bottom left, 1—bottom right, 2—top left, and 3—top right). Blue‐shaded areas represent the 95% CI from model simulations, and the median of the observed data is shown as blue open circles connected by splines.
**Figure S3:** Item characteristic curves showing the probability of each biomarker falling into ordered categories from 0 to 3 as a function of (a) the first latent variable (ψ_1_, blood pressure‐related dimension) and (b) the second latent variable (ψ_2_, metabolic dimension). Rows represent the seven biomarkers: systolic blood pressure (sBP), diastolic blood pressure (dBP), low‐density lipoprotein (LDL), high‐density lipoprotein (HDL), triglycerides (TG), glycated hemoglobin (HbA1c), and body mass index (BMI). Red lines represent the model‐predicted probabilities, and gray shaded areas represent the generalized additive model (GAM) smooth of the observed data.
**Figure S5:** Item characteristic curves (ICCs) from the unidimensional IRT model for each cardiometabolic biomarker. Each panel displays the probability of observing each response category: 0 (P1), 1 (P2), 2 (P3), and 3 (P4), as a function of the latent variable (ψ), representing overall cardiometabolic dysfunction. Biomarkers included are triglycerides (TG), glycated hemoglobin (HbA1c), body mass index (BMI), systolic blood pressure (sBP), diastolic blood pressure (dBP), low‐density lipoprotein cholesterol (LDL), and high‐density lipoprotein cholesterol (HDL). Higher values of ψ correspond to greater cardiometabolic dysfunction.
**Table S6:** Final parameter estimates for the baseline and longitudinal MIRT model.
**Table S4:** Summary table of the predictive performance of latent variables for the mortality transition, compared to the base model without covariates.

**Data S1:** NONMEM code for the final IRT model.
