# Supplementary material for: Longitudinal Modeling of Cardiometabolic Dysfunction in Patients With Type 2 Diabetes Using Item Response Theory
Source: CPT Pharmacometrics Syst Pharmacol. 2026 Oct 15;15(11):e70349. doi: 10.1002/psp4.70349 (PMC13738796; doi:10.1002/psp4.70349)
Supplement: Supplementary file 1 [file PSP4-15-e70349-s001.docx]

Supplementary material

Figure S1. Change in Akaike Information Criterion (AIC) and Bayesian Information Criterion (BIC) relative to the unidimensional model for baseline IRT models with one to seven latent dimensions. The dashed line at zero represents the reference unidimensional model.

Figure S2. Categorical Visual Prediction Checks (VPCs) for the final longitudinal MIRT model. Each sub-panel shows the model-predicted probability of falling into each of the four ordered categories (i.e., 0 - bottom left, 1 - bottom right, 2 - top left, and 3 - top right). Blue-shaded areas represent the 95% CI from model simulations, and the median of the observed data is shown as blue open circles connected by splines.

Figure S3. Item characteristic curves showing the probability of each biomarker falling into ordered categories from 0 to 3 as a function of (a) the first latent variable ($\psi$*_1_*, blood pressure-related dimension) and (b) the second latent variable ($\psi$*_2_*, metabolic dimension). Rows represent the seven biomarkers: systolic blood pressure (sBP), diastolic blood pressure (dBP), low-density lipoprotein (LDL), high-density lipoprotein (HDL), triglycerides (TG), glycated hemoglobin (HbA1c), and body mass index (BMI). Red lines represent the model-predicted probabilities, and grey shaded areas represent the generalized additive model (GAM) smooth of the observed data.

Table S4. Summary table of the predictive performance of latent variables for the mortality transition, compared to the base model without covariates

| Model | ΔOFV | C_index_ | ΔC_index_ | Brier_score_ | ΔBrier_score_ |
| --- | --- | --- | --- | --- | --- |
| Base (reference) | 0 | 0.6722 | 0 | 0.04445 | 0 |
| $\psi$_1_ | -2028 | 0.6735 | 0.0013 | 0.04439 | -0.00006 |
| $\psi$_2_ | -1145 | 0.6875 | 0.0153 | 0.04393 | -0.00052 |
| $\psi$_1_ and $\psi$_2_ | -2317 | 0.6797 | 0.0075 | 0.04426 | -0.00019 |

Figure S5. Item characteristic curves (ICCs) from the unidimensional IRT model for each cardiometabolic biomarker. Each panel displays the probability of observing each response category: 0 (P1), 1 (P2), 2 (P3), and 3 (P4), as a function of the latent variable ($\psi$), representing overall cardiometabolic dysfunction. Biomarkers included are triglycerides (TG), glycated hemoglobin (HbA1c), body mass index (BMI), systolic blood pressure (sBP), diastolic blood pressure (dBP), low-density lipoprotein cholesterol (LDL), and high-density lipoprotein cholesterol (HDL). Higher values of $\psi$ correspond to greater cardiometabolic dysfunction.

Table S6. Final parameter estimates for the baseline and longitudinal MIRT model

|  | **Biomarker** | $Discrimination, \psi_{1}$  **Estimate (RSE%)** | $Discrimination, \psi_{2}$  **Estimate (RSE%)** | *Difficulty 1 **  **Estimate (RSE%)** | *Difficulty 2 **  **Estimate (RSE%)** | *Difficulty 3 **  **Estimate (RSE%)** |
| --- | --- | --- | --- | --- | --- | --- |
| ***Baseline model parameters*** | |  |  |  |  |  |
|  | sBP | 2.22 (1.7) | 0 (FIX) | −1.75 (1.0) | 1.05 (1.3) | 0.88 (1.2) |
|  | dBP | 1.84 (2.2) | 0.82 (2.8) | −1.40 (1.1) | 1.04 (1.3) | 1.21 (1.0) |
|  | LDL | 0.18 (12.7) | 0.13 (13.8) | − 9.04 (10.8) | 5.71 (11.0) | 13.02 (10.8) |
|  | HDL | 0 (FIX) | 0.86 (6.1) | −2.89 (4.6) | 1.66 (5.0) | 2.22 (4.7) |
|  | TG | 0.12 (20.7) | 0.66 (7.9) | − 0.20 (11.7) | 5.24 (7.2) | — |
|  | HbA1c | 0.10 (13.9) | 0.33 (6.2) | − 4.18 (5.7) | 3.40 (5.8) | 3.13 (5.7) |
|  | BMI ^a^ | 0.17 (9.2) | 0.59 (3.7) | − 2.86 (3.5) | 2.79 (3.4) | 2.09 (3.5) |
|  | ^a^ – the additional BMI-specific intercept was estimated of 0.01 (RSE 48.9%) | | | | | |
|  | **Covariate effects*** | | |  |  |  |
|  | Age at T2D diagnosis, year^-1^ | 0.02 (4.9) | -0.07 (5.1) |  |  |  |
|  | Women vs men | — | -0.43 (6.3) |  |  |  |
|  | * – age at T2D diagnosis was centered at 61 years. Men were used as the reference group for the sex covariate. | | | | | |
| ***Longitudinal model parameters*** | | | | | | |
|  | LDL | 0.29 (7.7) | 0.001 (3.4) | -7.49 (7.5) | 6.2 (7.6) | 13.37 (7.6) |
|  | HDL | 0 (FIX) | 2.53 (3.0) | -1.98 (1.4) | 1.03 (1.8) | 1.32 (1.6) |
|  | HbA1c | 0.14 (18.9) | 0.40 (3.9) | -2.73 (5.7) | 2.81 (6.0) | 3.2 (5.9) |
|  | **Disease progression parameters** | | |  |  |  |
|  | Shared slope | -0.028 (9.1) | -0.029 (9.3) |  |  |  |
|  | Post-baseline offset | -0.10 (7.6) | -0.22 (6.2) |  |  |  |
|  | HbA1c-specific slope — 0.45 (4.6) | | | | | |

* Difficulty parameters are reported as incremental values.
