# Supplementary material for: Longitudinal Modeling of Cardiometabolic Dysfunction in Patients With Type 2 Diabetes Using Item Response Theory
Source: CPT Pharmacometrics Syst Pharmacol. 2026 Oct 15;15(11):e70349. doi: 10.1002/psp4.70349 (PMC13738796; doi:10.1002/psp4.70349)
Supplement: Supplementary file 2 [file PSP4-15-e70349-s002.docx]

**NONMEM code for the final IRT model**

$SIZES	LIM6=4000 LTH=-1000 DIMNEW=-10000

$PROBLEM	entry data with 6 4-categorical items and 1 3-categorical item, two dimensions

$INPUT	ID DAT2=DROP TIME DV ITEM AGE SEX BAGE MDV

$DATA	data.csv IGNORE=@

$PRED

; -----------------------------------------------constants to select model type-------------------------------------------

IF(NEWIND.NE.2) THEN

BTIME=0

TOT=0

NUM=0

ENDIF

PSI_MODEL=0

UNDEF=0

OC1=1	; ordered categorical [0,3]

OC2=2	; ordered categorical [0,2]

MODEL=0

; --------------------------------------------------assignment of covariates-------------------------------------------------

COVAGE1 = THETA(44)

COVAGE2 = THETA(45)

BPMed1 = THETA(46)

COVSEX2 = THETA(47)

; -----------------------------------------------assignment of item parameters------------------------------------------

; -----------------------------------------------sBP------------------------------------------

IF(ITEM.EQ.1) THEN

MODEL=OC1

DIS1=THETA(1)

DIS2=THETA(2)

DIF1=THETA(3)

DIF2=THETA(4)

DIF3=THETA(5)

GUE=THETA(6)

; -----------------------------------------------dBP-----------------------------------------------

ELSE IF(ITEM.EQ.2) THEN

MODEL=OC1

DIS1=THETA(7)

DIS2=THETA(8)

DIF1=THETA(9)

DIF2=THETA(10)

DIF3=THETA(11)

GUE=THETA(12)

; -----------------------------------------------LDL -----------------------------------------------

ELSE IF(ITEM.EQ.3) THEN

MODEL=OC1

DIS1=THETA(13)

DIS2=THETA(14)

DIF1=THETA(15)

DIF2=THETA(16)

DIF3=THETA(17)

GUE=THETA(18)

;-----------------------------------------------HDL -----------------------------------------------

ELSE IF(ITEM.EQ.4) THEN

MODEL=OC1

DIS1=THETA(19)

DIS2=THETA(20)

DIF1=THETA(21)

DIF2=THETA(22)

DIF3=THETA(23)

GUE=THETA(24)

;-----------------------------------------------TG -----------------------------------------------

ELSE IF(ITEM.EQ.5) THEN

MODEL=OC2

DIS1=THETA(25)

DIS2=THETA(26)

DIF1=THETA(27)

DIF2=THETA(28)

GUE=THETA(29)

; -----------------------------------------------HbA1c -----------------------------------------------

ELSE IF(ITEM.EQ.6) THEN

MODEL=OC1

DIS1=THETA(30)

DIS2=THETA(31)

DIF1=THETA(32)

DIF2=THETA(33)

DIF3=THETA(34)

GUE=THETA(35)

; -----------------------------------------------BMI -----------------------------------------------

ELSE IF(ITEM.EQ.7) THEN

MODEL=OC1

DIS1=THETA(36)

DIS2=THETA(37)

DIF1=THETA(38)

DIF2=THETA(39)

DIF3=THETA(40)

GUE=THETA(41)

ENDIF

;---------------------------------------------------The latent variables model----------------------------------------------

TVPSI1=THETA(42)

TVPSI2=THETA(43)

EFF1 = 0 ;offset on PSI1

EFF2 = 0 ;offset on PSI2

IF(TIME.GT.0) THEN

EFF1=THETA(48)

EFF2 = THETA(50)

ENDIF

SL1 = THETA(49)

SL2 = THETA(51)

PSI1=TVPSI1+ETA(1)+(BAGE-61)*COVAGE1+EFF1+SL1*(AGE-BAGE) ; Latent variable 1

PSI2=TVPSI2+ETA(2)+(BAGE-61)*COVAGE2+EFF2+SL2*(AGE-BAGE) ; Latent variable 2

IF(SEX.EQ.2) PSI2=TVPSI2+ETA(2)+(BAGE-61)*COVAGE2+EFF2+SL2*(AGE-BAGE)+COVSEX2

;-------------------------ordered categorical data model with 4 levels: [0,3]---------------------------

IF(MODEL.EQ.OC1) THEN

DIFG1=DIF1

DIFG2=DIFG1+DIF2

DIFG3=DIFG2+DIF3

PGE1=GUE+(1-GUE)/(1+EXP((DIS1*(DIFG1-PSI1))+(DIS2*(DIFG1-PSI2))))

PGE2=GUE+(1-GUE)/(1+EXP((DIS1*(DIFG2-PSI1))+(DIS2*(DIFG2-PSI2))))

PGE3=GUE+(1-GUE)/(1+EXP((DIS1*(DIFG3-PSI1))+(DIS2*(DIFG3-PSI2))))

P0=1-PGE1

P1=PGE1-PGE2

P2=PGE2-PGE3

P3=PGE3

PPRED=P1*1 + P2*2 + P3*3

SDPRED=SQRT(P0*(0-PPRED)**2 + P1*(1-PPRED)**2 + P2*(2-PPRED)**2 + P3*(3-PPRED)**2)

PGE1LV1=GUE+(1-GUE)/(1+EXP(DIS1*(DIFG1-PSI1)))

PGE2LV1=GUE+(1-GUE)/(1+EXP(DIS1*(DIFG2-PSI1)))

PGE3LV1=GUE+(1-GUE)/(1+EXP(DIS1*(DIFG3-PSI1)))

P0LV1=1-PGE1LV1

P1LV1=PGE1LV1-PGE2LV1

P2LV1=PGE2LV1-PGE3LV1

P3LV1=PGE3LV1

PPREDLV1=P1LV1*1 + P2LV1*2 + P3LV1*3

SDPREDLV1=SQRT(P0LV1*(0-PPREDLV1)**2 + P1LV1*(1-PPREDLV1)**2 + P2LV1*(2-PPREDLV1)**2 + P3LV1*(3-PPREDLV1)**2)

PGE1LV2=GUE+(1-GUE)/(1+EXP(DIS2*(DIFG1-PSI2)))

PGE2LV2=GUE+(1-GUE)/(1+EXP(DIS2*(DIFG2-PSI2)))

PGE3LV2=GUE+(1-GUE)/(1+EXP(DIS2*(DIFG3-PSI2)))

P0LV2=1-PGE1LV2

P1LV2=PGE1LV2-PGE2LV2

P2LV2=PGE2LV2-PGE3LV2

P3LV2=PGE3LV2

PPREDLV2=P1LV2*1 + P2LV2*2 + P3LV2*3

SDPREDLV2=SQRT(P0LV2*(0-PPREDLV2)**2 + P1LV2*(1-PPREDLV2)**2 + P2LV2*(2-PPREDLV2)**2 + P3LV2*(3-PPREDLV2)**2)

ENDIF

;-------------------------ordered categorical data model with 3 levels: [0,2]---------------------------

IF(MODEL.EQ.OC2) THEN

DIFG1=DIF1

DIFG2=DIFG1+DIF2

PGE1=GUE+(1-GUE)/(1+EXP((DIS1*(DIFG1-PSI1))+(DIS2*(DIFG1-PSI2))))

PGE2=GUE+(1-GUE)/(1+EXP((DIS1*(DIFG2-PSI1))+(DIS2*(DIFG2-PSI2))))

P0=1-PGE1

P1=PGE1-PGE2

P2=PGE2

PPRED=P1*1 + P2*2

SDPRED=SQRT(P0*(0-PPRED)**2 + P1*(1-PPRED)**2 + P2*(2-PPRED)**2)

PGE1LV1=GUE+(1-GUE)/(1+EXP(DIS1*(DIFG1-PSI1)))

PGE2LV1=GUE+(1-GUE)/(1+EXP(DIS1*(DIFG2-PSI1)))

P0LV1=1-PGE1LV1

P1LV1=PGE1LV1-PGE2LV1

P2LV1=PGE2LV1

PPREDLV1=P1LV1*1 + P2LV1*2

SDPREDLV1=SQRT(P0LV1*(0-PPREDLV1)**2 + P1LV1*(1-PPREDLV1)**2 + P2LV1*(2-PPREDLV1)**2)

PGE1LV2=GUE+(1-GUE)/(1+EXP(DIS2*(DIFG1-PSI2)))

PGE2LV2=GUE+(1-GUE)/(1+EXP(DIS2*(DIFG2-PSI2)))

P0LV2=1-PGE1LV2

P1LV2=PGE1LV2-PGE2LV2

P2LV2=PGE2LV2

PPREDLV2=P1LV2*1 + P2LV2*2

SDPREDLV2=SQRT(P0LV2*(0-PPREDLV2)**2 + P1LV2*(1-PPREDLV2)**2 + P2LV2*(2-PPREDLV2)**2)

ENDIF

IF(MODEL.EQ.OC1.AND.DV.LE.0) P=P0

IF(MODEL.EQ.OC1.AND.DV.EQ.1) P=P1

IF(MODEL.EQ.OC1.AND.DV.EQ.2) P=P2

IF(MODEL.EQ.OC1.AND.DV.GE.3) P=P3

IF(MODEL.EQ.OC2.AND.DV.LE.0) P=P0

IF(MODEL.EQ.OC2.AND.DV.EQ.1) P=P1

IF(MODEL.EQ.OC2.AND.DV.GE.2) P=1-P0-P1

;-------------------------Response probability prediction and residual-----------

IF(P.LT.1E-8) P=1E-8 ; To protect for P->0

IF(P.GT.1) P=1-1E-8 ; To protect for P->1

Y=-2*LOG(P)

PWRES = (DV - PPRED) / SDPRED

PWRESLV1 = (DV - PPREDLV1) / SDPREDLV1

PWRESLV2 = (DV - PPREDLV2) / SDPREDLV2

IF (MODEL.EQ.UNDEF) THEN

PWRES=0

P=0

Y=0

ENDIF

;-------------------------------------------------------simulation code-------------------------------------------------------

IF(ICALL.EQ.4) THEN

IF(MODEL.EQ.OC1) THEN

CALL RANDOM(2, R)

SDV=0

IF(R.LT.PGE1) SDV=1

IF(R.LT.PGE2) SDV=2

IF(R.LT.PGE3) SDV=3

ELSE IF(MODEL.EQ.OC2) THEN

CALL RANDOM(2, R)

SDV=0

IF(R.LT.PGE1) SDV=1

IF(R.LT.PGE2) SDV=2

ENDIF

DV=SDV

IF(ITEM.EQ.8) THEN

IF(NEWIND.NE.2) THEN

DV=0

TTE=0

OTTE=0 ;count for previous event

CALL RANDOM(2,R)

USUR=R

ENDIF

IF(OTTE.EQ.0) THEN

IF(USUR.GT.SUR) THEN

IF(TIME.LE.8) THEN

DV=1

TTE=1

OTTE=1

ENDIF

ELSE

IF(TIME.GT.8) THEN

DV=0

TTE=1

OTTE=1

ENDIF

ENDIF

ENDIF

ENDIF

IF(ITEM.EQ.9) DV = -99

ENDIF

$ESTIMATION METHOD=COND LAPLACE -2LL MAXEVAL=9999

$THETA …

$OMEGA …
